# How Educators’ Self-Construal Shapes Teacher Training: Navigating from Autism Awareness to Stigma

**DOI:** 10.1007/s10803-025-06788-x

**Published:** 2025-03-19

**Authors:** Mahmut Serkan Yazıcı, İsmail Karsantık

**Affiliations:** 1 Department of Special Education, Faculty of Education, Recep Tayyip Erdogan University, Rize, Turkey; 2 Department of Educational Administration, Faculty of Education, Recep Tayyip Erdogan University, Rize, Turkey

**Keywords:** Autism, Self-construal, Stigma, Teacher education, Inclusive education, Culture

## Abstract

This study examined how self-construal, a key aspect of cultural structure, influences teacher training to improve autism awareness and reduce stigma. It explored autism awareness, self-construal, and stigma levels among potential educators, as well as the relationships between these factors. The study also investigated the mediating role of self-construal in the link between autism awareness and stigma. The study included 1031 potential educators—individuals with no teaching experience with students with ASD but likely to work with them in the future. Participants were selected through purposeful sampling. Data were collected using a demographic form, Autism Awareness Scale, Self-Construal Scale, and Stigma Scale. The study found that both autonomous and relational self-construals significantly impacted autism awareness and stigma. These cultural factors influenced how potential educators perceive and respond to ASD. The analysis highlighted the mediating role of self-construal between autism awareness and stigma. The study concluded that self-construal, as a cultural element, plays a significant role in shaping potential educators’ approaches to autism awareness and stigma reduction. It is recommended that teacher training programs incorporate cultural factors like self-construal to complement efforts in enhancing autism awareness and reducing stigma, ensuring that potential educators’ cultural structures do not outweigh their professional qualifications in interactions with students with ASD.

Autism Spectrum Disorder (ASD) is a neurodevelopmental condition characterized by restricted and repetitive behaviors and interests, as well as deficits in social interaction and communication skills in early childhood (American Psychiatric Association [APA], 2013). Current data from the Centers for Disease Control and Prevention (CDC, 2024a) indicates a global increase in ASD diagnoses across all races, ethnic groups, and socioeconomic levels. The increase in the number of individuals with ASD worldwide is reflected in schools, with a growing number of students with ASD in general education settings in recent years (Al Jaffal, 2022; Wittwer et al., 2024). Therefore, it is crucial for all educators to be prepared to support students with ASD, as achieving high-quality inclusive education otherwise becomes challenging (Bolourian et al., 2021; Donath et al., 2023).

When preparing educators to educate students with ASD, one of the most fundamental factors is autism awareness (Autism Education Trust, 2020a), as it is essential for understanding, accepting, and appreciating individuals with ASD (Autism Education Trust, 2020b; Hamilton, 2012). For example, common characteristics related to social skills observed in individuals with ASD (e.g., a tendency to be alone, difficulty establishing relationships with peers) may differ from those of neurotypical individuals (Baio et al., 2018). Although these differences may lead others to perceive individuals with ASD as antisocial, this perception is inaccurate (Samenow, 2020). Therefore, it is critical for educators to understand ASD and their students with ASD accurately in order to implement practices that can improve the students’ social skills. In fact, according to the All-Party Parliamentary Group for Autism report (2017), six out of 10 students with ASD state that having an educator who understands them is the most important factor in making school a better place for them. Thus, educators’ autism awareness is a key element in the educational process for students with ASD.

In the development of autism awareness, several factors, such as knowledge about ASD (Golson et al., 2022), media influence (Pesonen et al., 2021), personal experiences related to ASD (Gillespie-Lynch et al., 2017), and culture (de Leeuw et al., 2020), may play a role. Among these elements, culture is particularly important to consider because it has been emphasized, from its initial definition to the present, that culture is an umbrella concept that influences many aspects (e.g., beliefs, values, attitudes, and behaviors) in the lives of individuals within a social group (Causadias, 2020; Tylor, 1871). Therefore, culture can also affect the development of educators’ autism awareness (Wang, 2024).

To better understand the role of culture in educators’ autism awareness in this research, it is useful to examine culture in more detail through Hofstede’s Cultural Dimensions Theory (Hofstede, 1980). One of the six dimensions addressed by this theory is Individualism-Collectivism, which is directly relevant to the focus of the current study (Self-construal). Hofstede evaluated societies in terms of self-construal and was interested in how integrated and committed individuals in these societies were to their communities. He further classified societies into individualistic and collectivistic categories.

Although culture exists in dynamic, multi-layered structures (Kemmelmeier & Kühnen, 2012), with varying cultural orientations—such as African Americans, Asian Americans, and Latino Americans in the United States (Vargas & Kemmelmeier, 2013)—it is widely recognized that national cultures in most Western countries (including the United States, Canada, Great Britain, and many Western European nations) tend to be more individualistic. In contrast, the national culture of many Eastern countries (such as Japan, China, and South Korea) is typically more collectivistic (Hofstede et al., 2010). Markus and Kitayama (1991) explained that, in addition to the various differences between these two cultural structures, the element that forms the basis of these differences is self-construal, which refers to how an individual perceives themselves in relation to others. When considering this difference in more detail, in individualistic societies where autonomous self-construal is dominant, individuals tend to define themselves as unique. In contrast, in collectivistic societies where relational self-construal is prevalent, individuals typically explain themselves based on their relationships, roles, and connections within the group. For example, when individuals need to define themselves, those with an autonomous self-construal provide answers focused more on themselves, such as “I am creative,” whereas those with a relational self-construal provide answers based on their relationships, connections, and roles in society, such as “I am a mother” (Giacomin & Jordan, 2017). Thus, the influence of others and society is more significant in collectivistic cultures, where relational self-construal is dominant, compared to individualistic cultures, where autonomous self-construal is prevalent (Liu et al., 2021).

The cultural differences mentioned above can affect every individual, including individuals with ASD, and can bring both advantages and risks for them (Ennis-Cole et al., 2013; Yazıcı, 2023). For example, while separation from others is more common in individualistic cultures where autonomous self-construal is dominant, interpersonal connectedness is stronger in collectivistic cultures where relational self-construal is prevalent (Markus & Kitayama, 1991). Because of this effect of self-construal, the individual characteristics of individuals with ASD may be more acceptable, as being unique is encouraged in individualistic cultures (Markus & Kitayama, 1991). While this is an advantage for individuals with ASD, it also presents a risk, as loneliness resulting from individualistic culture can negatively affect mental health (DiJulio et al., 2018; Scott et al., 2004). On the other hand, due to the interconnectedness of individuals in a collectivistic culture, the formation of social networks that can support the development of social skills among individuals with ASD can be seen as an advantage (Yazıcı, 2018). However, within these social networks, stigmatization—expressed as social disapproval and discrimination of a person—may occur (APA, 2018). Because in collectivistic cultures, individuals are evaluated based on social harmony rather than their own individuality (Markus & Kitayama, 1991; Young, 2023). This situation can lead to stigmatization, which is one of the most serious risks present in collectivistic cultures due to the characteristics of individuals (Atherton et al., 2023; Papadopoulos, 2016). Such stigma can negatively affect the well-being of individuals with ASD by creating situations that significantly impair their lives, such as mental and physical health problems and exclusion from social life (Turnock et al., 2022). All these factors suggest that the role of cultural elements (e.g., self-construal) may be critical in understanding ASD and its awareness.

In light of the above statements, it is clear that cultural structures should not be ignored when raising autism awareness. This consideration is particularly important for educators, who have significant duties and responsibilities in the educational lives of students with ASD, such as preparing and implementing individualized education programs (e.g. Ministry of National Education, 2018). Therefore, in addition to understanding the extent to which the training provided enhances their autism awareness, it is also essential to consider how this awareness develops within the framework of their cultural structures. Because culture influences many aspects of individuals, such as beliefs, behaviors, and attitudes (Coleman et al., 2024), it may be believed that elements associated with ASD are interpreted correctly within a particular cultural norm. However, this interpretation may not always be accurate. For example, there is a belief in Chinese society that a child who speaks late may be more intelligent than their peers in the future (Sun et al., 2013). While one of the common symptoms of ASD is delayed speech (CDC, 2024b), this symptom may be overlooked or misinterpreted by those who hold this belief in China. This issue can also be observed among professionals working with individuals with ASD in collectivistic cultures. For instance, in South Korea, some clinicians may deliberately make alternative diagnoses for students with ASD to avoid stigmatization (Hughes, 2011). Therefore, educators’ autism awareness may manifest differently in their school life due to cultural factors. In this context, it is important to understand the role of educators’ cultural characteristics in the training process aimed at increasing their autism awareness.

## Research Significant, Aim and Questions

This research was conducted based on three main elements. The first element is autism awareness, which is a fundamental aspect that should be developed in educators who interact with students with ASD (Autism Education Trust, 2020b). The second element, stigma, is known to be one of the most important factors that autism awareness studies aim to reduce (e.g., Kitchin & Karlin, 2022); therefore, it was included in the study. To understand the cultural effects of the relationship between these two elements, self-construal is included in the research, as it is considered one of the important indicators of individuals’ cultural structures (Markus & Kitayama, 1991).

To understand why these three elements were addressed in the research, it is useful to consider existing studies. Globally, efforts such as creating autism-friendly environments, promoting positive media portrayals, providing community training, and organizing events like World Autism Awareness Day aim to reduce stigma toward individuals with ASD while enhancing autism awareness (Turnock et al., 2022; United Nations, 2024). Many studies have also examined the link between autism awareness and stigma (e.g. Kim et al., 2024). However, there is limited research on the role of culture in this relationship. One reason for this may be that most existing studies have been conducted in Western countries with individualistic cultures, where many autism-related tools and research were originally developed (Henrich et al., 2010). However, their interpretation may differ in Eastern countries, which may not fully account for cultural differences (de Leeuw et al., 2020). Therefore, to explore cultural roles, research is needed in collectivist countries. Thus, although this study addresses autism awareness and stigma in a manner similar to other studies, it distinguishes itself by exploring self-construal as a cultural factor in this relationship. An important distinguishing feature of the current study is its focus on potential educators—those who have no teaching experience with students with ASD but are likely to work with students diagnosed with ASD in the future. These individuals are expected to play a crucial role in shaping the lives of students with ASD, making their preparation and understanding particularly significant for the study.

In line with the reasons stated above, the main purpose of the current study was to examine the mediating role of self-construal in the effect of potential educators’ autism awareness levels on stigmatization tendencies. Additionally, the study aimed to determine the levels of potential educators’ autism awareness, self-construal, and stigma, as well as to examine the relationships between these levels. To achieve these aims, the following research questions were formulated:

1. What are the levels of potential educators’ autism awareness, self-construal, and stigmatization?
2. What is the relationship between potential educators’ autism awareness, self-construal, and stigmatization?
3. What is the mediating role of self-construal in the effect of potential educators’ autism awareness levels on stigmatization?

## Method

In line with the objectives of the current study, a correlational research model within a quantitative research framework was employed. Correlational studies are particularly valuable for exploring the strength and direction of relationships, which makes them well-suited to inform educational practices and interventions (Fraenkel et al., 2023).

### Population and Sample

To achieve the purpose of this current study, purposive sampling was employed to recruit potential educator participants. Purposive sampling involves the deliberate selection of participants based on specific, pre-determined criteria relevant to the research objectives (Nyimbili & Nyimbili, 2024; Patton, 1990). In this study, the criterion for participation was being a potential educator with no prior experience working with students with ASD, but with the potential to gain such experience in the future. This included general education teachers, pre-service teachers in the faculty of education, and pre-service teachers in pedagogical formation training, who had graduated from non-education programs and were preparing for the teaching profession.

While determining the sample size, Nicolaou and Masoner (2013) suggested the appropriate sample size for structural equation modelling (SEM) with 28 degrees of freedom as 382. Based on this, 1164 potential educators were invited to participate in the study, comprising 902 pre-service teachers studying at a state university and 262 general education teachers from different public schools. However, not all invited participants participated in the study. A total of 1031 individuals took part, with 868 being pre-service teachers enrolled in higher education institutions in Türkiye during the 2023–2024 academic year, and the remaining 163 being currently employed general education teachers in Turkish schools. Table 1 presented the demographic characteristics of the participants.

**Table 1:** Demographic characteristics of the participants

Variable	Groups	Frequency (f)	Percentage (%)
Gender	Female	782	75.8
	Male	249	24.2
Education level	High School	568	55.1
	Undergraduate	425	41.2
	Postgraduate	38	3.7
Age	18–24	748	72.6
	25–34	136	13.2
	35–44	112	10.9
	45–54	35	3.4
Professional ladders	1st Year Students in the Faculty of Education	189	18.3
	2nd Year Students in the Faculty of Education	181	17.6
	3rd Year Students in the Faculty of Education	167	16.2
	4th Year Students in the Faculty of Education	161	15.6
	Graduated from a faculty other than the Faculty of Education and is currently involved in the pedagogical formation certificate program	170	16.5
	Graduate from the Faculty of Education or holder of a pedagogical formation certification	163	15.8
Status of qualification	Teacher	163	15.8
	Pre-service teacher (undergraduate student in the Faculty of Education)	698	67.7
	In the process of training within the pedagogical formation certificate program	170	16.5
Professional experience	No professional experience yet	738	71.6
	Less than 1 year	101	9.8
	1–5 years	59	5.7
	6–10 years	39	3.8
	11–15 years	40	3.9
	More than 15 years	54	5.2

### Data Collection Instruments

Data were obtained using several instruments: A demographic information form, the ‘Self-Construal Scale’ developed by Singelis (1994) and further validated by Singelis et al. (1995), referred to as the INDCOL scale, which was adapted into Turkish by Wasti and Eser Erdil (2007); the ‘Stigmatization Scale’ developed by Yaman and Güngör (2013); and the ‘Autism Awareness Scale’ developed by Yazıcı and Karsantık (2023).

#### Demographic Information Form

Demographic data of the participants were collected using a demographic information form prepared by the authors, which consisted of six questions. The purpose of this form was to obtain information about the participants’ gender, age, education level, professional ladders, status of qualification, and professional experience.

#### The Self-Construal Scale

The Self-Construal Scale (Wasti & Eser Erdil, 2007) was a two-dimensional structure comprising 30 items. The scale employed a seven-point Likert scale, with statements ranging from “strongly disagree” to “strongly agree.” It consisted of two dimensions: “individualism” and “collectivism,” with 15 items allocated to each dimension. The total score for each subscale could be obtained by summing the scores assigned on the seven-point Likert scale. The maximum score for both subscales was 105, while the minimum score was 15. The scores for the individualism and collectivism subscales were used to quantify the value (individualism or collectivism) that serves the self in various life domains. The Cronbach’s alpha internal consistency coefficient was found to be 0.63 for the individualism subscale and 0.72 for the collectivism subscale. In the current study, the Cronbach’s alpha internal consistency coefficient was calculated to be 0.70 for the individualism subscale and 0.69 for the collectivism subscale.

#### Stigmatization Scale

The Stigmatization Scale (Yaman & Güngör, 2013) was a four-dimensional instrument consisting of 22 items, designed to assess various aspects of stigmatization. Constructed using a five-point Likert format, respondents indicated their level of agreement with statements ranging from “strongly disagree” to “strongly agree.” The scale permitted a minimum score of 22 and a maximum score of 110, and notably, it did not include any reverse-coded items. The items were categorized into four dimensions: six items measuring discrimination and exclusion, six items focusing on labeling, five items assessing psychological health, and five items addressing prejudice.

The internal consistency of the scale was evaluated using Cronbach’s alpha, yielding a coefficient of 0.84 for the total scale. The subscales demonstrated varying levels of consistency, with coefficients of 0.77 for discrimination and exclusion, 0.68 for labeling, 0.66 for psychological health, and 0.54 for prejudice. Furthermore, the Confirmatory Factor Analysis (CFA) results indicated strong model fit indices, including a Root Mean Square Error of Approximation (RMSEA) of 0.064, a Comparative Fit Index (CFI) of 0.93, an Incremental Fit Index (IFI) of 0.93, a Normed Fit Index (NFI) of 0.90, a Relative Fit Index (RFI) of 0.89, an Adjusted Goodness of Fit Index (AGFI) of 0.88, and a Goodness of Fit Index (GFI) of 0.91. The Cronbach’s alpha coefficients for the stigma scale in the current study were calculated as 0.77 for the discrimination and exclusion subscale, 0.50 for the labeling subscale, 0.62 for the psychological health subscale, and 0.46 for the prejudice subscale, indicating varying reliability across dimensions.

#### Autism Awareness Scale

The Autism Awareness Scale (Yazıcı & Karsantık, 2023) was a seven-dimensional structure comprising 32 items. It employed a five-point Likert scale, with statements ranging from “strongly disagree” to “strongly agree.” The total variance explained by the Autism Awareness Scale was 62% (α = 0.92). The first subscale, designated “Limited Interests and Repetitive Behaviors in Autism,” consisted of six items (α = 0.90). The second subscale, also comprising six items, was titled “Social Interaction and Communication in Autism” (α = 0.91). The third subscale, entitled “Causes of Autism,” included six items (α = 0.84). The fourth subscale, titled “Friendships in Autism,” comprised four items (α = 0.91). The fifth subscale, “Basic Elements in the Intervention Process in Autism,” consisted of four items (α = 0.83). The sixth subscale, “Physical Characteristics in Autism,” included three items (α = 0.79). The seventh subscale was designated as “Early Warning Signs of Autism,” comprising three items (α = 0.78).

Upon analysis of the current study data, it was evident that the Cronbach’s alpha values for the subscales were as follows: 0.85 for the first subscale, 0.68 for the second subscale, 0.82 for the third subscale, 0.38 for the fourth subscale, 0.88 for the fifth subscale, 0.88 for the sixth subscale, and 0.85 for the seventh subscale, with an overall total scale value of 0.47. Taber (2018) stated that Cronbach’s alpha values ranging from 0.45 to 0.90 are considered acceptable for demonstrating internal consistency and reliability. However, in cases where the scale was intended to capture a multifaceted construct, lower alpha values may be observed, suggesting that the items may not all be measuring the same underlying trait (McNeish, 2018). This perspective lends further support to the notion that while a Cronbach’s alpha falls below the conventional threshold, it may nevertheless offer valuable insights into the reliability of the scale within its specific context. (Lyu et al., 2020).

The results of the Confirmatory Factor Analysis (CFA) indicated that the Chi-square/standard deviation (χ²/sd) value was 2.1, the Root Mean Square Error of Approximation (RMSEA) was 0.58, the Standardized Root Mean Square Residual (SRMR) was 0.06, the Normed Fit Index (NFI) was 0.87, the Non-Normed Fit Index (NNFI) was 0.92, the Comparative Fit Index (CFI) was 0.93, the Goodness of Fit Index (GFI) was 0.85, and the Adjusted Goodness of Fit Index (AGFI) was 0.82.

### Data Collection and Analysis

Prior to initiating the data collection phase of the study, approvals were obtained from the University Ethics Commission and the relevant institutional authorities from which data would be collected to conduct the research and utilize the scales. Subsequently, a pilot study was conducted with 10 non-participants (five general education teachers and five pre-service teachers) to test the research form, which included the research information form, participant consent form, and data collection tools. Based on the grammatical feedback received, the form was finalized with necessary adjustments to enhance its comprehensibility.

Afterward, time planning was conducted by contacting the participants’ institutions to arrange the data collection process. The institutions were visited, and the participants were informed about the research in person. The research form, which was prepared in both online and printed formats, was presented according to the preferences of the participants and their institutions. They chose the online version due to its advantages, which included not taking up class time and allowing completion of the form outside the institution. The link to the research form was shared with participants via their individual or institutional emails, in accordance with their preferences. The time to complete the form ranged from a minimum of eight minutes to a maximum of fifteen minutes, with an average time of twelve minutes. Data collection was completed successfully within the eight-month timeframe of the study. After data collection ended, the data from 1,031 participants were securely stored in an encrypted digital environment for further analysis.

In the initial stages of the analysis process, the dataset was evaluated to determine whether it met the unidirectional and multidirectional normality assumptions. This entailed an examination of the skewness and kurtosis values of the data, as well as a visual representation of the data through a Q-Q graph. It was observed that the kurtosis-skewness values of the individualism and collectivism dimensions of the self-construal scale exhibited a normal distribution, with values ranging from − 0.01 to 0.28 and from − 0.00 to 0.07, respectively. The kurtosis and skewness values for the subscales of the Stigmatization Scale, namely discrimination and exclusion, labeling, psychological health, and prejudice, were align with the normal range, with values ranging from 1.0 to 0.97, 0.46 to 0.92, 0.07 to -0.15, and from 0.02 to 0.16, respectively. In terms of autism awareness scale, values with regard to the limited interest and repetitive behaviors subscale (-0.26 to − 0.31), physical characteristics subscale (0.09 to − 0.22), friendships in autism subscale (-0.25 to 0.31), social interaction and communication subscale (-. 05 to 1.1), causes of autism subscale (-0.33 to 0.55), basic elements in the intervention process in autism subscale (-0.16 to 0.12), early warning signs of autism subscale (-0.63 to 0.10) were found to be within expected range. Skewness and kurtosis values for the autism awareness scale were between − 0.005 and 0.33, while the values for the total score of the stigmatization scale were between 0.32 and 0.14. As stated by Tabachnick et al. (2019), kurtosis-skewness values between − 1.5 and + 1.5 are indicative of a normally distributed dataset. Furthermore, the dispersion of the data in the Q-Q graph, which assumes the form of an ellipse around the line with an angle of 45 degrees, also corroborates the assumption of normality. The presence of multicollinearity was investigated through the examination of correlation values between variables. The correlation values of 0.85 indicate that there is no multicollinearity problem (Pallant, 2020). The arithmetic mean was interpreted with regard to the following ranges: 1.00–1.80 was considered as “very low”, 1.81–2.60 as “low”, 2.61–3.40 as “medium”, 3.41–4.20 as “high” and 4.21–5.00 as “very high”. In the interpretation of the correlation analysis, a low level of relationship was defined as a range between 0.00 and 0.30, a medium level as a range between 0.31 and 0.70, and a high level as a range between 0.71 and 1.00. The first question of the study was investigated using the arithmetic mean, the second question employed Pearson Moment Correlation analysis, and the third question was addressed through structural equation modeling.

The initial step involved testing the assumptions of normal distribution, multicollinearity, and linearity. By detecting multicollinearity, the models can be refined, ensuring that the relationships between the predictors and the dependent variable are accurately represented, thus enhancing the interpretability and robustness of the results (Harefa et al., 2023; Ningthoujam et al., 2023). Accordingly, the skewness and kurtosis values fall within the acceptable range of ± 1.5, as defined by Tabachnick et al. (2019). Specifically, the skewness value was between − 0.005 and 1.049, while the kurtosis value was between − 0.317 and 1.194. To test the multicollinearity assumption, the variance inflation factor (VIF) was calculated. A VIF value of 10 or above indicates the presence of multicollinearity within the data (Neter et al., 1996). The VIF values obtained from the current study (1.01 < VIF < 1.03) indicated the absence of multicollinearity.

A structural equation model was established to test the mediating role of self-construal in the effect of potential educators’ autism awareness levels on stigmatization. To further validate the structural equation model with mediating variables, it was essential to establish significant relationships between exogenous and endogenous variables, as highlighted by Baron and Kenny (1986). In this context, the number of bootstrap samples was determined to be 1000 (Arbuckle, 2007) to test the significance of the direct and indirect effects of the observed and latent variables in the model. The causal-steps model proposed by Baron and Kenny (1986) and the Sobel test are regarded as insufficiently robust for mediation analysis; thus, the bias-corrected bootstrap is recommended as a superior approach for detecting mediation (Preacher & Hayes, 2008). The standard bootstrap can, in certain circumstances, yield estimates that are not representative of the data. This is particularly likely to occur in small samples or when the underlying distribution of the data is non-normal, resulting in unreliable statistical inferences (Fritz et al., 2012). Conversely, the bias-corrected bootstrap method has been shown to adjust for this limitation by incorporating corrections during the resampling process (Fritz & MacKinnon, 2007), thereby enhancing the robustness of the estimates and improving the coverage probabilities of confidence intervals. Consequently, the bias-corrected bootstrap emerged as a valuable instrument for enhancing the precision and dependability of statistical analyses.

In contemporary mediation analysis, the distinction between complete and partial mediation is no longer considered valid, as it is based on specific empirical research whose results are influenced by sample size and methodological constraints. (Fritz & MacKinnon, 2007; Zhao et al., 2010). Additionally, Preacher and Hayes (2008) highlight the limitations of the Baron and Kenny (1986) approach, advocating for methods that focus on estimating indirect effects without the need for a significant direct effect. To ascertain the mediating role of self-construal, the procedures proposed by Zhao et al. (2010) were followed. Once the mediation effect of the direct relationship between X and M has been identified, further classifications can be made, namely complementary mediation, competitive mediation, indirect-only mediation, or direct-only nonmediation.

## Results

This section presented the findings related to the research questions. First, the findings regarding the participants’ perceptions of self-construal, stigmatization, and autism awareness were displayed in Table 2.

**Table 2:** Participants’ perceptions of self-construal, stigmatization and autism awareness

Variables		Minimum	Maximum	Mean	Std. Error	Std. Deviation	Level
Self-construction	Individualism	28	105	3.47	0.32	10.56	High
	Collectivism	32	105	3.17	0.32	10.52	Moderate
Stigmatization	Discrimination and exclusion	6	24	1.57	0.10	3.26	Very Low
	Labeling	6	30	2.28	0.10	3.36	Low
	Psychological health	5	23	2.34	0.10	3.35	Low
	Prejudice	5	24	2.84	0.09	3.00	Moderate
	*Total score for Stigmatization*	24	88	2.23	0.31	10.11	Low
Autism awareness	Limited interests and repetitive behaviors in autism	6	30	2.77	0.16	5.23	Moderate
	Physical characteristics in autism	3	15	2.98	0.08	2.74	Moderate
	Friendships in autism	4	20	3.48	0.09	3.11	High
	Social interaction and communication in autism	6	30	3.38	0.11	3.55	Moderate
	Causes of autism	6	30	3.44	0.13	4.19	High
	Basic elements in the intervention process in autism	4	20	3.40	0.10	3.22	High
	Early warning signs of autism	3	15	4.07	0.07	2.31	High
	*Total score for Autism Awareness Scale*	72	125	3.13	0.23	7.67	Moderate

In the interpretation of the data, the mean between 1.00 and 1.79 points was considered to indicate a very low perception level, between 1.80 and 2.59 points to indicate a low perception level, between 2.60 and 3.39 points to indicate a medium perception level, between 3.40 and 4.19 points to indicate a high perception level, and between 4.20 and 5.00 points to indicate a very high perception level. Upon examination of Table 2, it became evident that the participants’ perceptions of self-construal were notably high in the individualism self-construal dimension ($\:\stackrel{-}{X}$= 3.47) and moderately high in the collectivism self-construal dimension ($\:\stackrel{-}{X}$= 3.17). The participants demonstrated minimal perceptions of discrimination and exclusion ($\:\stackrel{-}{X}$=1.57), labeling ($\:\stackrel{-}{X}$=2.28), psychological health ($\:\stackrel{-}{X}$=2.34), prejudice ($\:\stackrel{-}{X}$=2.84), and stigmatization in general ($\:\stackrel{-}{X}$=2.23) as indicated by the mean scores. The participants demonstrated a medium level of awareness regarding limited interest and repetitive behaviors in autism awareness ($\:\stackrel{-}{X}$= 2.77), a medium level of awareness concerning physical characteristics in autism ($\:\stackrel{-}{X}$= 2.98), a high level of awareness pertaining to friendships in autism ($\:\stackrel{-}{X}$= 3.48), and a medium level of awareness related to social interaction and communication in autism ($\:\stackrel{-}{X}$= 3.38), a high level of awareness with regard to the causes of autism ($\:\stackrel{-}{X}$= 3.44), a high level of awareness concerning the basic elements in the intervention process in autism ($\:\stackrel{-}{X}$= 3.40), a high level of awareness related to early warning signs of autism ($\:\stackrel{-}{X}$= 4.07), and a medium level of autism awareness in an overall sense ($\:\stackrel{-}{X}$= 3.13).

The purpose of Table 3 was to determine whether there was a significant relationship between self-construal, stigmatization, and autism awareness. To this end, the table below presented the results of a correlation analysis conducted on the total scores and subscales of the participants’ perceptions of self-construal, stigmatization, and autism awareness.

**Table 3:** Pearson product moment correlation coefficient table representing the relationship between participants’ perceptions of self-construal, stigmatization, and autism awareness

	1a	1b	1c	1d	1e	1f	1 g	2a	2b	2c	2d	3a	3b	1	2
Limited interests and repetitive behaviors in autism (1a)	1														
Physical characteristics in autism (1b)	0.085^**^	1													
Friendships in autism (1c)	0.065^*^	0.591^**^	1												
Social interaction and communication in autism (1d)	0.264^**^	0.347^**^	0.497^**^	1											
Causes of autism (1e)	0.115^**^	0.494^**^	0.727^**^	0.575^**^	1										
Basic elements in the intervention process in autism (1f)	0.065^*^	0.706^**^	0.782^**^	0.490^**^	0.628^**^	1									
Early warning signs of autism (1 g)	0.011	0.104^**^	0.346^**^	0.281^**^	0.358^**^	0.268^**^	1								
Discrimination and exclusion (2a)	0.079^*^	0.043	− 0.061^*^	− 0.010	− 0.011	− 0.054	− 0.169^**^	1							
Labeling (2b)	0.124^**^	0.036	− 0.051	0.002	− 0.008	− 0.028	− 0.124^**^	0.516^**^	1						
Psychological health (2c)	0.150^**^	0.061	− 0.025	0.036	0.003	− 0.008	− 0.112^**^	0.502^**^	0.544^**^	1					
Prejudice (2d)	0.121^**^	− 0.006	− 0.069^*^	0.047	0.000	− 0.040	− 0.108^**^	0.334^**^	0.453^**^	0.475^**^	1				
Individualism (3a)	0.034	0.023	0.057	0.061^*^	0.062^*^	0.051	0.119^**^	− 0.090^**^	− 0.026	− 0.079^*^	− 0.005	1			
Collectivism (3b)	0.098^**^	0.095^**^	0.047	0.148^**^	0.084^**^	0.083^**^	0.045	0.007	0.065^*^	0.140^**^	0.086^**^	0.167^**^	1		
Total score for Autism awareness scale (1)	0.745^**^	0.045	0.316^**^	0.584^**^	0.156^**^	0.329^**^	0.398^**^	− 0.049	0.016	0.048	0.040	0.070^*^	0.101^**^	1	
Total score for stigmatization (2)	0.153^**^	0.044	− 0.066^*^	0.024	− 0.005	− 0.041	− 0.165^**^	0.761^**^	0.815^**^	0.816^**^	0.713^**^	− 0.066^*^	0.096^**^	0.017	1

*Significant at 0.05 level

**Significant at 0.001 level

Upon examination of Table 3, it became evident that there was a notable positive correlation between the total score on the Autism Awareness Scale and limited interest and repetitive behaviors subscale (*r* =.745; *p* <.001), friendships in autism subscale (*r* =.316; *p* <.001), social interaction and communication subscale (*r* =.584; *p* <.001), causes of autism subscale (*r* =.156; *p* <.001), basic elements in the autism intervention process subscale (*r* =.329; *p* <.001), early warning signs of autism (*r* =.398; *p* <.001), individualism (*r* =.070; *p* <.05) and collectivism (*r* =.101; *p* <.001).

Significant correlations were identified between the total score of the Stigmatization Scale and several other subscales, including limited attention and repetitive behaviors in autism (*r* =.153; *p* <.001), friendships in autism (*r*=-.066; *p* <.05), early warning signs of autism (*r*=-.165; *p* <.001), discrimination and exclusion (*r* =.761; *p* <.001), labeling (*r* =.815; *p* <.001), psychological health (*r* =.816; *p* <.001), prejudice (*r* =.713; *p* <.001), individualism (*r*=-.066; *p* <.001) and collectivism (*r* =.096; *p* <.001).

Upon examination of Table 4, it became evident that the regression coefficients associated with the participants’ perceptions of autism awareness with regard to their perceptions of the collectivism (β = 0.074; *p* >.05), individualism (β = 0.069; *p* >.05), and stigmatization (β = − 0.065; *p* >.05) were statistically insignificant. Furthermore, the collectivism perception of the participants was found to have a significant positive predictive coefficient (β = 0.134; *p* <.05) on their perceptions of stigmatization, while the individualism perception was found to have a significant negative predictive coefficient (β = − 0.093; *p* <.05) on the same variable. The results indicated that participants’ perceptions of autism awareness were positively predicting the dimensions of limited interest and repetitive behaviors in autism (β = 0.082), physical characteristics in autism (β = 0.625; *p* <.05), friendships in autism (β = 0.011; *p* <.05), social interaction and communication in autism (β = 0.552; *p* <.05), causes of autism (β = 0.775; *p* <.05), basic elements in the intervention process in autism (β = 0.830; *p* <.05), and early warning signs of autism (β = 0.373; *p* <.05). Stigmatization perceptions were found to positively predict discrimination and exclusion subscale (β = 0.657, *p* <.05), labeling subscale (β = 0.745, *p* <.05), psychological health subscale (β = 0.760, *p* <.05), and prejudice subscale (β = 0.594, *p* <.05).

**Table 4:** Regression weights between self-construal, stigmatization, and autism awareness

Relationships between variables			Regression Weights				S.*R*.W. Estimate
			Estimate	S.E	C.*R*.	*P*	
Collectivism	←	Autism awareness	1.824	1.073	1.700	0.08	0.074
Individualism	←	Autism awareness	1.691	1.041	1.624	0.10	0.069
Stigmatization	←	Autism awareness	− 0.324	0.222	-1.456	0.14	− 0.065
Stigmatization	←	Collectivism	0.027	0.007	3.843	***	0.134
Stigmatization	←	Individualism	− 0.019	0.007	-2.683	0.00	− 0.093
Limited interests and repetitive behaviors in autism	←	Autism awareness	1.000				0.082
Physical characteristics in autism	←	Autism awareness	4.003	1.591	2.516	0.01	0.625
Friendships in autism	←	Autism awareness	6.806	2.693	2.528	0.01	0.938
Social interaction and communication in autism	←	Autism awareness	4.554	1.759	2.589	0.01	0.552
Causes of autism	←	Autism awareness	7.579	3.002	2.524	0.01	0.775
Basic elements in the intervention process in autism	←	Autism awareness	6.248	2.473	2.526	0.01	0.830
Early warning signs of autism	←	Autism awareness	2.012	0.812	2.479	0.01	0.373
Discrimination and exclusion	←	Stigmatization	1.000				0.657
Labeling	←	Stigmatization	1.167	0.065	17.959	***	0.745
Psychological health	←	Stigmatization	1.189	0.066	18.091	***	0.760
Prejudice	←	Stigmatization	0.831	0.054	15.410	***	0.594

*Note*. S.R.W.= Standardised Regression Weights, S.E.: Estimation of Error, C.R.: Critical Ratio

^***^p <.001

**Fig. 1:**
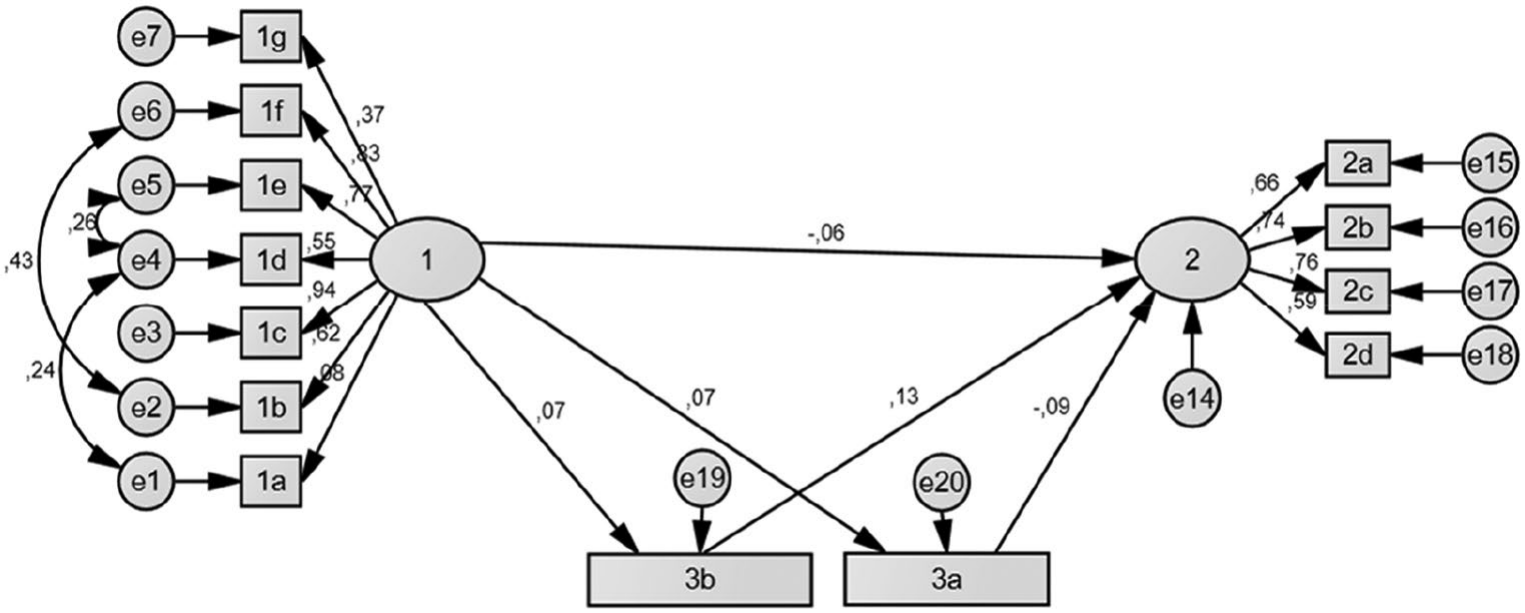
Structural equation model for the mediating effect of self-construal in the relationship between autism awareness and stigmatization. *Note*. 1 = autism awareness, 1a = limited interests and repetitive behaviors in autism, 1b = physical characteristics in autism, 1c = friendships in autism, 1d = social interaction and communication in autism, 1e = causes of autism, 1f = basic elements in the intervention process in autism, 1 g = early warning signs of autism, 2 = stigmatization, 2a = discrimination and exclusion, 2b = labeling, 2c = psychological health, 2d = prejudice, 3a = individualism, 3b = collectivism

The results of the structural equation model, which was constructed to investigate the mediating effect of self-construal on the relationship between autism awareness and stigmatization, were presented in Fig. 1. Upon examination of the model depicted in Fig. 1, it was evident that the moderator value demonstrating the strongest indirect effect (ab) was observed between the collectivism subscale and the psychological health subscale (ab = 0.10; *p* <.05). Conversely, the moderator value revealing the weakest indirect effect was identified between the individualism and the prejudice subscale (ab = 0.05; *p* <.05). Additionally, Table 5 provided a summary of the fit indices for the tested structural equation model.

**Table 5:** The fit values for the tested SEM model indicating the mediating role of self-construal in the relationship between autism awareness and stigmatization

Fit indices	Values for the tested structural equation model	Relatively strong value	Acceptable fit value	References
(*x*^2^/sd)	4.68	$\:\le\:$3	$\:\le\:$4–5	(Carmines & McIver, 1981)
RMSEA	0.06	$\:\le\:$0.05	0.06-0.08	Browne and Cudeck (1992)
SRMR	0.05	$\:\le\:$0.05	0.06-0.08	(Hu & Bentler, 1999)
CFI	0.95	$\:\ge\:$0.95	0.90-0.94	(McDonald & Marsh, 1990)
TLI	0.94	$\:\ge\:$0.95	0.90-0.94	(Bentler & Bonett, 1980)
GFI	0.96	$\:\ge\:$0.95	0.90-0.94	(Joreskog & Sorbom, 1984)
AGFI	0.94	$\:\ge\:$0.90	0.80-0.89	(Joreskog & Sorbom, 1984)

Upon examining Table 5, it became evident that the fit indices of the model demonstrate a relatively strong and acceptable fit, indicating that the model adequately represented the relationships among the variables involved. Notably, fit values served not only to reinforce the adequacy of the model in capturing the interrelationships among the variables, but also to emphasise the necessity of distinctions between direct and indirect effects for the purpose of achieving a comprehensive understanding of the mediation process involved. The absence of significance in the direct effect would indicate the presence of indirect-only mediation. This classification was made in accordance with the insignificance of the direct effect (c’) of X on M. If c’ was deemed insignificant, it could be concluded that indirect-only mediation was established. The concept of indirect-only mediation was analogous to that of full mediation as proposed by Baron and Kenny (1986). Given that autism awareness was found to have no direct and significant effect on stigmatization (β= − 0.06; *p* >.05), it could be concluded that self-construal acted as a fully mediating variable. The coefficients for the direct and indirect effects, as determined using a 95% confidence interval, were presented in Table 6.

**Table 6:** Bootstrapping for the model indicating the mediating role of self-construal in the relationship between autism awareness and stigmatization

Paths	Direct effects	Indirect effect	95% CI
Autism awareness → Individualism	0.06^**^		[0.03, 0.15]
Autism awareness → Collectivism	0.07^**^		[0.00, 0.13]
Autism awareness → Stigmatization	− 0.06		[-0.13, 0.00]
Autism awareness → Self-Construal → Stigmatization		0.004^**^	[-0.00, 0.01]

^**^p <.001

The direct and indirect coefficients and the significance of the variables in the model were examined using bias-corrected bootstrap. The indirect path coefficient, which provides insight into the mediating role, was found to be significant (β = 0.004, CI = 95%, LB = − 0.008, UB = 0.01). Therefore, it was evident that self-construal played a mediating role in the relationship between autism awareness and stigmatization.

## Discussion

Within the scope of this research, it was found that the participating potential educators’ self-construal played an active role in the effect of their autism awareness levels on their stigmatization tendencies. The study showed that autonomous self-construal, along with the development of individuals’ autism awareness, makes the reduction of stigmatization tendencies more effective. On the other hand, this effect was indicated to be in the opposite direction for individuals with relational self-construal. In other words, as autism awareness increases, the self-concept of individuals with relational self-construal creates a barrier to the anticipated decrease in stigmatization tendencies.

Considering the findings, existing research shows that relational self-construal is more prominent in collectivistic cultures, and stigmatizing tendencies are more common in these societies compared to individualistic cultures (Ran et al., 2021; Sum et al., 2024). This cultural context may help explain the barriers created by relational self-construal within the scope of this research. However, it is important to note that addressing this barrier, linked to relational self-construal, is not straightforward in teacher education programs. Therefore, evaluating autism awareness training through the lens of participants’ self-construal may provide valuable insights into how cultural barriers affect teacher education.

A crucial aspect of autism awareness training involves understanding how resources on ASD are perceived by individuals from different cultural backgrounds. While ASD exists universally, it is often interpreted through Western-based frameworks, where autonomous self-construal predominates. Tools and resources for diagnosing and understanding ASD, such as screening and diagnostic tests, are primarily developed in North America and Europe (Williams, 2019). As a result, individuals in collectivistic cultures, such as those in Eastern countries, may interpret these resources differently from those in the West, potentially leading to misunderstandings. For example, the lack of eye contact is commonly viewed as a symptom of ASD in Western countries (CDC, 2024b). However, in some collectivistic cultures, avoiding eye contact is a sign of respect (Uono & Hietanen, 2015), which could influence how ASD is perceived. This mismatch between the cultural context of autism awareness programs and local norms may limit their effectiveness. In Türkiye, for instance, where relational self-construal is dominant (Göregenli, 1997; Hofstede et al., 2010), general autism awareness training may not fully align with the cultural values of educators, thereby hindering its impact. To improve the effectiveness of such programs, it is essential to adapt them to the cultural structures and values of the target audience. Ignoring these cultural differences could reduce the positive outcomes expected from autism awareness initiatives in educational settings. To better understand this, it would be useful to examine the autism awareness of potential educators within the scope of the research.

When the findings regarding participants’ autism awareness were examined, it was found that although they had a high level of awareness in some areas related to ASD (e.g., causes of ASD, early warning signs of ASD, elements in the intervention process in ASD, friendships in ASD), their overall autism awareness was at a moderate level. While it is positive that potential educators do not have a low level of autism awareness, it is crucial that they possess a high level of awareness across all aspects of autism (Autism Education Trust, 2020a). This is because a lack of comprehensive autism awareness among potential educators may introduce certain risks. One such risk could be related to the potential educator’s cultural background (e.g., Irvine, 2003). For instance, participants were found to have a moderate level of awareness regarding social interaction and communication in ASD. This moderate level of awareness could pose a potential risk to students with ASD, depending on the potential educator’s cultural context. For instance, a common sign of ASD is that individuals with ASD may show little interest in interacting with others (APA, 2013). If potential educators interpret this behavior as a lack of desire to interact with others at school, it may lead to misinterpretations of the behaviors of students with ASD. In particular, an educator with a relational self-construal, who values group harmony, may be more inclined to view the student with ASD as problematic within the group (Qu, 2019). Such misinterpretations could have a direct and negative impact on the school experiences of students with ASD. In this context, in addition to improving potential educators’ competencies regarding autism awareness, it is important that educational content be adapted to reflect their cultural norms. Otherwise, the influence of the educator’s cultural background may overshadow their professional skills in the educational process.

To understand how effectively educators’ cultural structures impact the educational process for students with ASD —particularly in the context of inadequate autism awareness—it is essential to evaluate potential educators’ stigmatization tendencies within the scope of this research. To understand how effectively educators’ cultural structures impact the educational process for students with ASD—particularly in the context of inadequate autism awareness—it is essential to evaluate potential educators’ stigmatization tendencies within the scope of this research. The study found that participants had a low tendency to stigmatize. As autism awareness increases, individuals’ stigmatization tendencies generally decrease (Kitchin & Karlin, 2022). However, in this study, the level of autism awareness was not high enough to significantly reduce stigma. In fact, no relationship was found between autism awareness and stigmatization tendencies. This unexpected finding suggests that factors beyond awareness might play a critical role in shaping stigmatization tendencies. While awareness provides information, it does not necessarily translate into attitude or behavior change, especially in cultural contexts where relational self-construal dominates. For example, in collectivist cultures like Türkiye, societal harmony and group norms often take precedence over individual knowledge, potentially limiting the impact of awareness initiatives (e.g., Mac Cárthaigh & López, 2020).

Furthermore, cultural values and beliefs, institutional policies, and media representations may act as mediators in reducing stigma (Arboleda-Flórez, 2002; Turnock et al., 2022). Among these, culture plays a dominant role, as it fundamentally shapes many aspects of human behavior (Aaron & Elizabeth, 2018). Similarly, in this research, culture appears to be the primary factor affecting the tendency to stigmatize. The participants’ autonomous self-construal, which was more dominant, may explain the low stigmatization tendency (Papadopoulos, 2016). Thus, it was the potential educators’ autonomous self-construal, rather than their autism awareness, that contributed to their low tendency to stigmatize. Although this is a positive finding, there is a potential risk if educators adhere too closely to their cultural frameworks when practicing their profession (Banks & Banks, 2019), as negative aspects of culture can be included in the process alongside positive features. For example, a teacher with a relational self-construal may form a stronger bond with their student with ASD than one with an autonomous self-construal; however, this bond might weaken if the teacher’s self-construal is autonomous (Yazıcı, 2018). These findings highlight the importance of addressing cultural factors and self-construal in understanding the complex relationship between autism awareness and stigmatization tendencies, particularly in collectivist societies.

Additionally, the current study demonstrates that interventions can be developed for potential educators’ self-construals to positively influence the cultural situations mentioned above. In this context, the participants’ self-construals reflect this, as the study was conducted in a country (Türkiye) where collectivist culture is dominant, and relational self-construal is prevalent among individuals (Göregenli, 1997; Hofstede et al., 2010). However, it was found that the majority of participants in this study exhibit characteristics of autonomous self-construal. Therefore, it can be inferred that teacher education can lead individuals toward a more autonomous structure (e.g., Fabela Cárdenas, 2012). This situation indicates that the self-construal of most potential educators differs from the general cultural norms of society. This difference demonstrates that, although national culture plays a dominant role in shaping individuals (Hofstede et al., 2010), cultural change is also possible (Varnum & Grossmann, 2017).

In addition to the general effect of teacher training on self-construal, the impact of developing autism awareness on self-construal is also significant in this study. As awareness of autism increases, it is found that the characteristics of individuals’ autonomous self-construal rise, along with their relational self-construal. This suggests that autism awareness is developed in conjunction with social norms in teacher education. This has both positive aspects and potential risks. For instance, as relational self-construal increases, “other-focused emotions,” defined as feelings centered on other individuals, may also develop, fostering emotional bonds and closeness with others (Markus & Kitayama, 1991). However, this situation may also have negative implications, such as fostering feelings of pity towards individuals with ASD (Yazıcı, 2023). Therefore, if the cultural changes occurring in the development of autism awareness within teacher education do not occur within the framework of a professional intervention, this may lead to the persistence of cultural risks alongside positive outcomes in the process.

### Implications for Practice

**Fig. 2:**
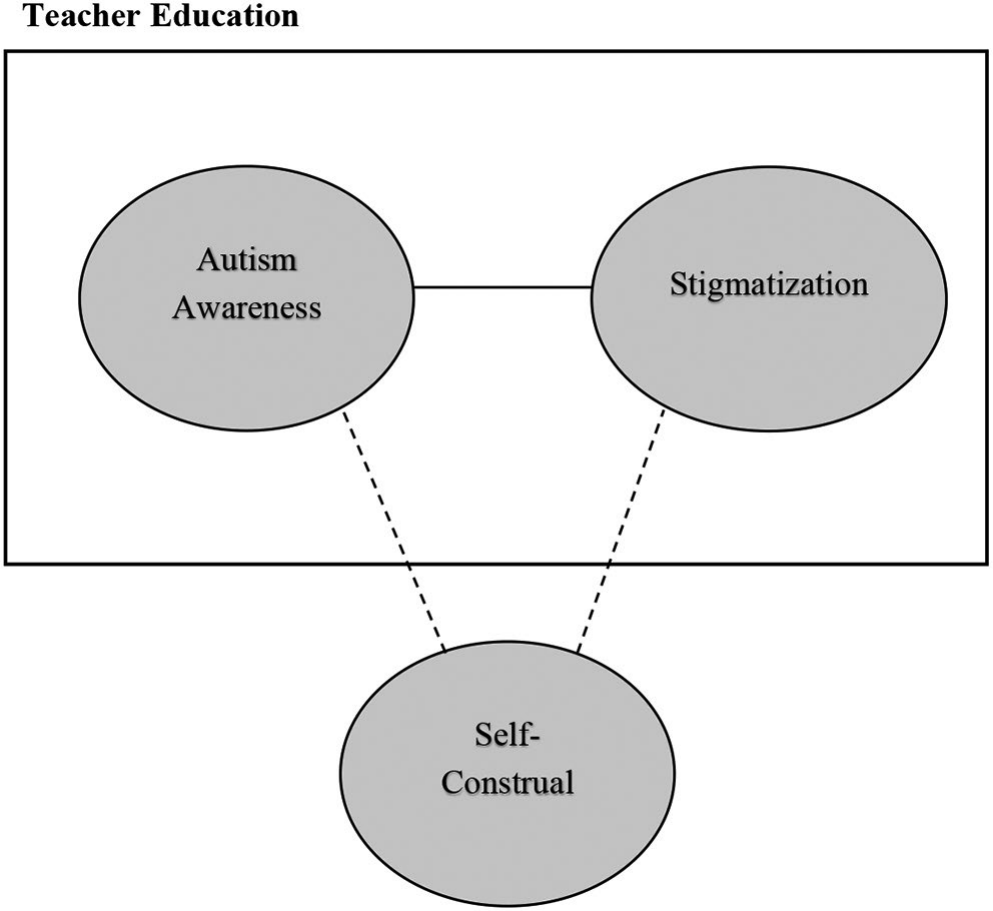
The teacher education process in which self-construal is overlooked

**Fig. 3:**
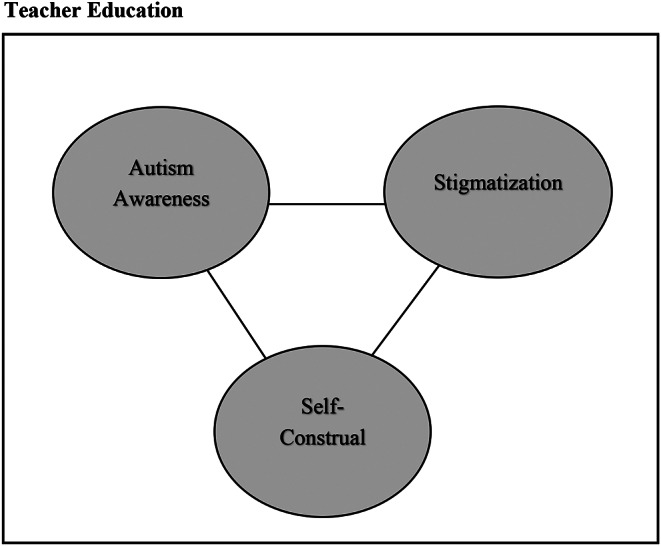
The educational process in which self-construal is incorporated into teacher education

Within the scope of the research, it was noted that the teacher training shown in Fig. 2 would increase the potential educator-based cultural risks associated with the process. Therefore, it is understood that the autism awareness expected to be developed, as well as the goal of reducing stigmatization tendencies, should be integrated into the teacher training process along with the potential educators’ self-construal, as illustrated in Fig. 3.

Therefore, when planning and conducting autism-related training in teacher education, potential educators’ cultural structures, such as self-construal, should be analyzed, and content and interventions for the educational process should be prepared accordingly. To achieve this, regardless of whether potential educators’ self-construals are relational or autonomous, the potential risks associated with their self-construals should be identified, and interventions should be developed to address these risks. Additionally, the advantages of self-construal should be taken into consideration, and their development and application within a professional framework should be supported in these interventions. Otherwise, insufficient awareness of students with ASD may cause educators’ cultural structures to influence students more dominantly than their professional roles.

Considering the above elements, several strategies can be implemented to apply the findings of the current study in teacher training programs. First, it may be beneficial to incorporate cultural elements, such as self-construal, into both pre-service and in-service teacher training content related to the education of students with ASD. Additionally, a specific training program focused on cultural elements can be developed for teacher education. This program could involve experience-based studies, guided by a mentor, as culture is directly related to individuals’ experiences with others (Brady et al., 2019). In this context, school-based inclusive mentoring within the framework of an experiential learning model (IEM), based on Kolb’s principles, could provide a valuable approach (Yazıcı & Uzuner, 2024). By integrating these strategies, teacher training programs can better equip potential educators with the cultural competence necessary to support students with ASD in diverse educational settings.

### Limitations and Future Research

Despite the large number of participants in the study, the generalizability of the findings is questioned regarding the representativeness of the sample, particularly because the current study was conducted in Türkiye. This limits the applicability of the findings to different demographic and cultural groups. Therefore, future research with a larger sample size and in different cultural contexts will help overcome this limitation and enhance the generalizability of the results. Another recommendation for future research is to develop data collection tools that are sensitive to the cultural nuances within specific contexts. This would help minimize limitations in data interpretation and provide a more nuanced understanding of the diverse cultural perspectives involved.

Future studies should also examine the effects of potential educators’ cultural structures (e.g., self-construal) on the educational process of students with ASD within teacher education, which includes elements to be developed (e.g., autism awareness) and elements to be reduced (e.g., stigmatization tendencies). To make these effects professional and qualified, it would be beneficial for future studies to focus on creating training programs that include professional interventions related to educators’ cultural structures.
